# Analysis of the small chromosomal *Prionium serratum* (Cyperid) demonstrates the importance of reliable methods to differentiate between mono- and holocentricity

**DOI:** 10.1007/s00412-020-00745-6

**Published:** 2020-11-09

**Authors:** M. Baez, Y. T. Kuo, Y. Dias, T. Souza, A. Boudichevskaia, J. Fuchs, V. Schubert, A. L. L. Vanzela, A. Pedrosa-Harand, A. Houben

**Affiliations:** 1 Leibniz Institute of Plant Genetics and Crop Plant Research (IPK), Gatersleben, 06466 Stadt Seeland, Germany; 2 Laboratory of Plant Cytogenetics and Evolution, Department of Botany, Federal University of Pernambuco, Recife, Pernambuco Brazil; 3 Laboratory of Cytogenetics and Plant Diversity, Department of General Biology, Center for Biological Sciences, State University of Londrina, Londrina, Paraná, 86057-970 Brazil; 4 KWS SAAT SE & Co. KGaA, 37574 Einbeck, Germany

**Keywords:** CENH3/CENPA, Centromere type, Holocentric chromosome, Evolution, Cyperids, Thurniceae

## Abstract

For a long time, the Cyperid clade (Thurniceae-Juncaceae-Cyperaceae) was considered a group of species possessing holocentromeres exclusively. The basal phylogenetic position of *Prionium serratum* (Thunb.) Drège (Thurniceae) within Cyperids makes this species an important specimen to understand the centromere evolution within this clade. In contrast to the expectation, the chromosomal distribution of the centromere-specific histone H3 (CENH3), alpha-tubulin and different centromere-associated post-translational histone modifications (H3S10ph, H3S28ph and H2AT120ph) demonstrate a monocentromeric organisation of *P. serratum* chromosomes. Analysis of the high-copy repeat composition resulted in the identification of two centromere-localised satellite repeats. Hence, monocentricity was the ancestral condition for the Juncaceae-Cyperaceae-Thurniaceae Cyperid clade, and holocentricity in this clade has independently arisen at least twice after differentiation of the three families, once in Juncaceae and the other one in Cyperaceae. In this context, methods suitable for the identification of holocentromeres are discussed.

**Supplementary Information:**

The online version contains supplementary material available at 10.1007/s00412-020-00745-6.

## Introduction

Centromeres are essential for the segregation of chromosomes to the daughter cells during mitosis and meiosis. Most organisms contain one single size–restricted centromere per chromosome (monocentromere) visible as a primary constriction during metaphase. However, in independent eukaryotic taxa, species with chromosomes without distinct primary constrictions visible at metaphase exist, which are referred to as holocentric. Instead, the spindle fibres attach along almost the entire poleward surface of the chromatids (reviewed in Schubert et al. (2020)). Holocentricity evolved at least 19 times independently in various green algae, protozoans, invertebrates, and different higher plant families (Dernburg 2001; Escudero et al. 2016; Melters et al. 2012). A phylogenetic analysis of more than 50,000 species demonstrated that holocentric species are most likely derived from their monocentric ancestors rather than the other way around (Escudero et al. 2016). In total, ~ 1.5–2.0% of flowering plants are likely to have holocentric chromosomes (Bures et al. 2012). It is possible that holocentricity is even more common than reported so far, as the identification of the centromere type, especially in small-sized chromosomes, is challenging. Besides mono- and holocentric chromosomes, species with elongated monocentromeres were reported, such as *Pisum* and *Lathyrus* species (Neumann et al. 2012; Neumann et al. 2016) and the red fire ant *Solenopsis invicta* (Huang et al. 2016). Consequently, they were regarded as evolutionary intermediates via runaway expansion of their centromeres toward the development of holocentromeres (Huang et al. 2016).

One common explanation for the evolution of holocentric chromosomes is their putative advantage related to DNA double-strand breaks (Zedek and Bures 2018). The studies on artificial chromosomal rearrangements in various holocentric species showed that chromosome fragments retaining centromere activity are transmitted during mitosis and meiosis (Jankowska et al. 2015). Comparisons of diversification rates between monocentric and holocentric sister clades in animals and plants did not detect an increase in diversification in holocentric species (Marquez-Corro et al. 2018). Nevertheless, these analyses depend on the correct identification of the centromere type in a large number of lineages.

Because holocentric taxa are often embedded within broader phylogenetic lineages possessing monocentric chromosomes, it is thought that holocentric chromosome organisation originated from the monocentrics and that this transition occurred independently in multiple phylogenetic lineages (Melters et al. 2012). However, the factors that induced this transition and its mechanisms are currently unknown. Investigations of the changes associated with the transition from monocentric to holocentric chromosome organisation are, in theory, most informative when phylogenetically closely related species that differ in the centromere type are compared.

In angiosperms, holocentric chromosomes have been confirmed in some dicot species, e.g. in the genus *Cuscuta* L., subgenus *Cuscuta* (Convolvulaceae) (Oliveira et al. 2020) and in a few species within the genus *Drosera* L. (Droseraceae) (Sheikh et al. 1995). Also, in monocots, for example, in the genus *Luzula* DC (Juncaceae) (Heckmann et al. 2013) and *Rhynchospora* Vahl. (Cyperaceae) (Marques et al. 2015; Ribeiro et al. 2017) holocentricity occurs. These last two families belong to the Cyperid clade (Thurniceae-Juncaceae-Cyperaceae), which was originally considered to share holocentric chromosomes as a synapomorphic feature (Greilhuber 1995; Judd et al. 2016; Melters et al. 2012). However, exceptions have been reported in the genus *Juncus* L., in which four species exhibited primary constrictions (Guerra et al. 2019). It suggests that this synapomorphy of the Cyperid clade is uncertain.

Aiming to improve the understanding on the origin and evolution of the holocentricity within the Cyperid clade, we studied the centromere organisation of *Prionium serratum* (L.f.) Drège (Thurniceae), a species phylogenetically situated at the base of the Cyperid clade (Silva et al. 2020) (Suppl. Fig. 1, Hochbach et al. 2018; Semmouri et al. 2019). The South African monocotyledonous plant genus *Prionium* E. Mey is an old, species-poor lineage which split from its sister genus about 26.1 million years ago (Kumar et al. 2017). *P. serratum* is suspected to be holocentric, as it is closely related to the families Juncaceae and Cyperaceae. Supported was this assumption by the fact that this species has a low genomic GC content, as it is typically described for holocentric species (Smarda et al. 2014). Furthermore, Zedek et al. (2016) observed no significant increase in the proportion of G2 nuclei after gamma irradiation of *P. serratum*, differing from the situation found in monocentric species.

To ascertain the centromere type of *P. serratum*, we determined the chromosomal distribution of the centromere-specific histone H3 (CENH3) protein and alpha-tubulin fibres. In addition, antibodies specific for the cell cycle–dependent pericentromeric phosphorylation of histone H3 (H3S10ph, H3S28ph) and histone H2A (H2AT120ph) were employed to distinguish between a mono- or holocentric chromosome structure. In monocentric plants, immunostaining of mitotic chromosomes with antibodies against H3S10ph and H3S28ph typically results in a specific labelling of the pericentromere only. In contrast, in holocentric plants, immunolabelling with the same antibodies produces a uniform staining of condensed chromosomes, due to the chromosome-wide distribution of the pericentromere (Gernand et al. 2003). The cell cycle–dependent phosphorylation of histone H2A at position threonine 120 is associated with active centromeres (Demidov et al. 2014; Dong and Han 2012). Contrary to the expectation, a monocentromeric organisation of the chromosomes was found. The analysis of the high-copy repeat composition resulted in the identification of two centromere-localised satellite repeats. In addition, a DNA replication behaviour was found typical for small genome monocentric species. The data are discussed in the context of centromere evolution in Cyperids and concerning the suitability of available methods to identify holocentromeres.

## Materials and methods

### Plant material

Individuals of *Prionium serratum* (L.f.) Drège collected in western Cape (Cape Town, South Africa; TE2016_413) and provided by the Herrenhäuser Gardens (Hannover, Germany, IPK herbarium 70142) and the Botanical Garden Halle (Halle, Germany) were grown in a greenhouse of the Leibniz Institute of Plant Genetics and Crop Plant Research (IPK Gatersleben, Germany).

### Flow cytometric genome size measurement

For nuclei isolation, roughly 0.5 cm^2^ of fresh leaf tissue was chopped together with equivalent amounts of leaf tissue of one of the internal reference standards, *Raphanus sativus* var. ‘Voran’ (Gatersleben genebank accession number: RA 34; 1.11 pg/2C) or *Lycopersicon esculentum* var. ‘Stupicke Rane’ (Gatersleben genebank accession number: LYC 418; 1.96 pg/2C), in a petri dish using the reagent kit ‘CyStain PI Absolute P’ (Sysmex) following the manufacturer’s instructions. The resulting nuclei suspension was filtered through a 50-μm filter mesh (CellTrics, Sysmex) and measured either on a CyFlow Space (Sysmex) or on a BD Influx cell sorter (BD Biosciences). The absolute DNA content (pg/2C) was calculated based on the values of the G1 peak means and the corresponding genome size (Mbp/1C), according to Dolêzel et al. (2003).

### DNA/RNA extraction and sequencing

Genomic DNA was extracted from *P. serratum* leaves using the DNeasy Plant Mini Kit (Qiagen) and sequenced using the HiSeq 2500 system (Illumina, CA) at low coverage. RNA was extracted from root meristems and prepared for paired-end sequencing on Illumina HiSeqX (Illumina, CA) by Novogene (Beijing, China).

### In silico repeat analysis

The repetitive proportion of the genome was analysed by the RepeatExplorer pipeline (Novak et al. 2013), implemented within the Galaxy/Elixir environment (https://repeatexplorer-elixir.cerit-sc.cz/). Low-coverage genomic paired reads were filtered by quality with 95% of bases equal to or above the quality cut-off value of 10 and interlaced. Clustering was performed with a minimum overlap of 55% and a similarity of 90%. Protein domains were identified using the tool Find RT Domains in RepeatExplorer pipeline (Novak et al. 2013). Searches using databases (GenBank) were performed and graph layouts of individual clusters were examined interactively using the SeqGrapheR program (Novak et al. 2013).

The number of analysed reads was 2,752,532 comprising in total ~ 276 Mbp, corresponding to 0.82× genome coverage. All clusters representing at least 0.01% of the genome were manually checked, and their automated annotation was corrected if necessary. The size of the annotated clusters was used to characterise and quantify the genome proportion of the high-copy repeats. To reconstruct the conserved monomer sequence of the tandem repeats, three independent runs were performed using the TAREAN (TAndem REpeat ANalyzer) tool implanted in RepeatExplorer (Novak et al. 2017).

### Repeat amplification, probe labelling and fluorescent in situ hybridisation

Satellite DNAs (satDNA) were PCR amplified with primers facing outwards of a repeat unit or directly synthesised as oligonucleotides with 5′-labelled fluorescence. Primers and oligonucleotides were designed from the most conserved region of the consensus sequences (Table 1). Forty nanograms of genomic DNA were used for all PCR reactions with 1× PCR buffer, 2 mM MgCl_2_, 0.1 mM of each dNTP, 0.4 μM of each primer, 0.025 U *Taq* polymerase (Qiagen) and water. PCR conditions were 94 °C 3 min, 30× (94 °C 1 min, 55 °C 1 min, 72 °C 1 min) and 72 °C 10 min. Amplicons and plasmid DNA of the 45S rDNA-containing clone pTa71 (Gerlach and Bedbrook 1979) were labelled with either Cy3, Atto488 or Atto550 fluorophores by a nick translation labelling kit (Jena Bioscience).

**Table 1:** High-copy satellite repeats of *P. serratum* and their corresponding chromosomal localisations

Satellites	Monomer length (in bp)	Genome proportion (in %)	BLAST	Chromosomal locations	Sequences of primers/oligo probes (5′-3′)
PsSat156a	156	0.05	-	Centromeric dot-like signals	F: ACATCGGGAGGACTCHTTG* R: ATTTTGGTTCCGGGAAAGTT
PsSat156b	156	1.40	-	Centromeric dot-like signals	F: AACTTTCCCCGAACCAAAAT R: CAGGTGTAGTTTGCCGAACA
PsSat306	306	2.70	-	One pairs of chromosomes	F: GGACATTGGGGTGGCTAGAG R: CGGTATTACACGGTCAAGAAGG
PsSat7	7	0.16	*Arabidopsis*-type telomere	Terminal of all chromosomes	5′-TAM-ACCCTAAACCCTAAACCC TAAACCCTAAACCCTAA
PsSat41	41	0.70	-	Two pairs of chromosomes	5′-TAM-AGGTCATTTTGCCTTGACA CCGGCC ATTGTGCATTTGACAC
PsSat311	311	0.17	-	Four pairs of chromosomes	F: CGGCAATCTACACATATGGTG R: GTTTGCTTAGCATGCCCACT
PsSat157	157	1.00	-	One pairs of chromosomes	F: GACTTTGACGAACGGATGGT R: GCAAACTTGATGTTGTGTTTGGC

*Nucleotide code H indicates A or C or T

-No sequence similarity detected

Mitotic chromosomes were prepared from root tips, pre-treated in 2 mM 8-hydroxyquinoline at 7 °C for 24 h and fixed in ethanol: acetic acid (3:1 v/v) for 2 to 24 h at room temperature and stored at − 20 °C. Fixed root tips were digested with 2% cellulose, 2% pectinase, and 2% pectolyase in citrate buffer (0.01 M sodium citrate dihydrate and 0.01 M citric acid) for 90 min at 37 °C and squashed in a drop of 45% acetic acid. Fluorescent in situ hybridisation was performed as described by Aliyeva-Schnorr et al. (2015). The hybridisation mix contained 50% (v/v) formamide, 10% (w/v) dextran sulphate, 2× SSC, and 5 ng/μl of each probe. Slides were denatured at 75 °C for 5 min, and the final stringency of hybridisation was 76%.

### RNA sequence analysis

We generated a total 15.6 Gbp of paired-end reads of 150 bp (around 52 million reads per end). Prior to mapping, all reads were preprocessed for quality control with FastQC, Galaxy version 0.72 (Andrews 2010). Subsequently, they were processed with the Trimmomatic program, Galaxy version 0.36.6 (Bolger et al. 2014) to trim adaptor contamination and low-quality sequences. As a result, 93.9% of high-quality sequences from total number were used for de novo transcriptome assembly with Trinity version 2.4.0. To evaluate the quality of assembly, its completeness and to remove poorly supported contigs, we applied Transrate v1.0.3 (Smith-Unna et al. 2016). The resulting dataset with 68,922 contigs was further processed by CD-HIT-EST, v. 4.6.8 program, using -c 0.95 -n 10 as parameters (Fu et al. 2012; Li and Godzik 2006) to cluster highly homologous sequences and remove redundant transcripts. Afterwards, the resulting file with 67,565 contigs was used to identify candidate coding regions within the transcript sequences (Transdecoder v. 5.3.0; http://transdecoder.github.io). RNAseq data are deposited in the European Nucleotide Archive under PRJEB39221 and genomic data are under NCBI SRX8683442. To identify a CENH3 candidate in the RNAseq data, we performed BLASTP, Galaxy Version 0.3.3 (Cock et al. 2015) using CENH3s from other monocotyledonous plants.

### Phylogenetic analysis

**Fig. 1:**
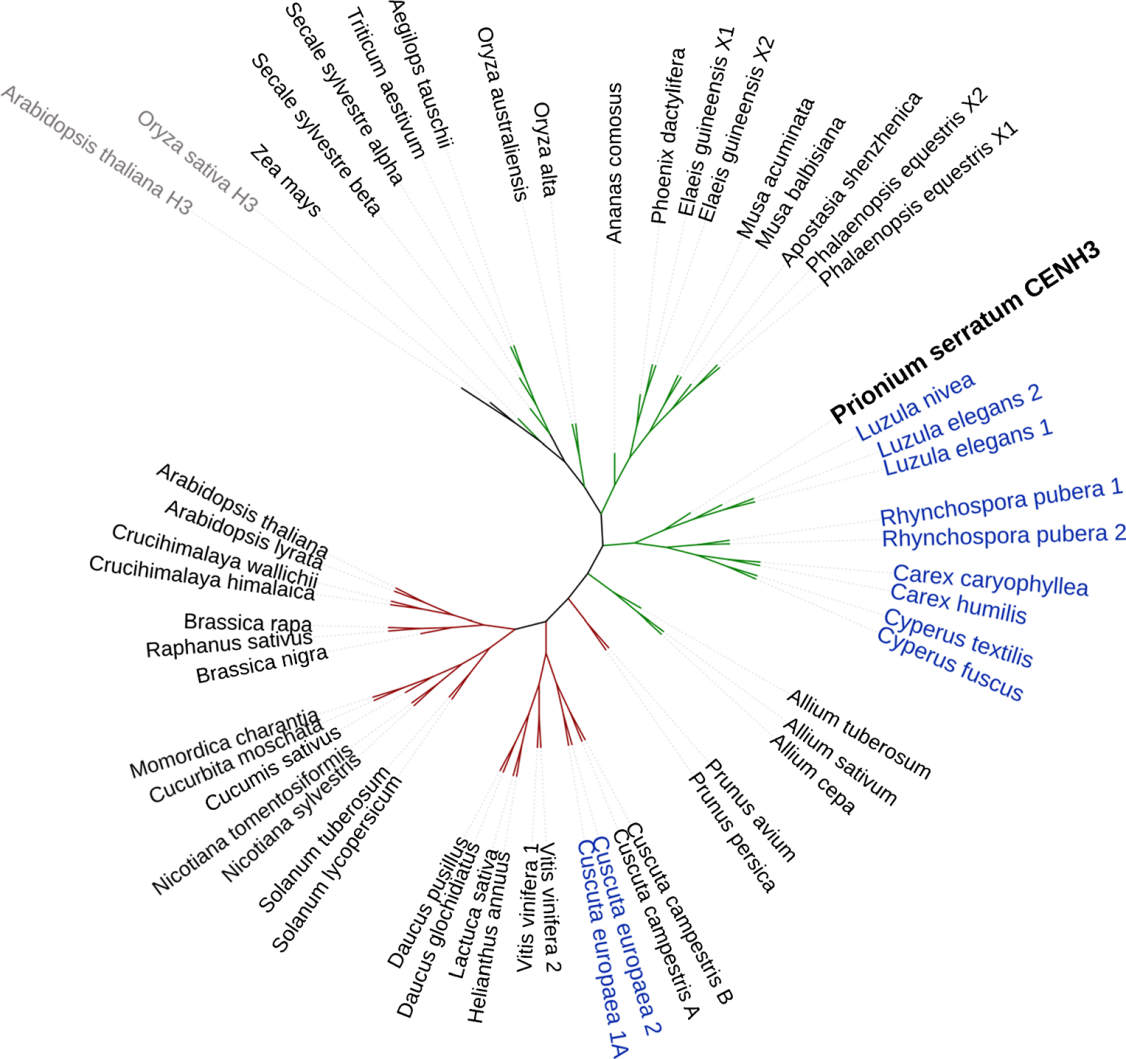
Phylogenetic relationship of CENH3 between *P. serratum* and other plant species. The green and red branch represent monocot and eudicot species, respectively. The blue node indicates the reported holocentric species, and the sequences of the canonical histone H3 used as outgroup are shown in grey node. The CENH3 sequence accession numbers are listed in Suppl. Table 1

The CENH3 sequence selected from *P. serratum* transcriptome dataset and those of other species downloaded from NCBI GenBank (see Fig. 1, Suppl. Table 1) were aligned with ClustalW implanted in MEGA X, using the default setting (Kumar et al. 2018; Thompson et al. 1994). The evolutionary relationship was inferred using the maximum likelihood method by the IQ-Tree web server (http://iqtree.cibiv.univie.ac.at) (Trifinopoulos et al. 2016). The built tree was visualised, labelled and exported by Interactive Tree Of Life (iTOL, https://itol.embl.de/) (Letunic and Bork 2007, 2019).

### Indirect immunostaining

The PsCENH3: RVKHFSNKAVSRTKKRIGSTR-c peptide was used for the production of polyclonal antibodies in rabbits. LifeTein (www.lifetein.com) performed the peptide synthesis, immunisation of rabbits and peptide affinity purification of antisera. Mitotic preparations were made from root meristems fixed in paraformaldehyde and Tris buffer (10 mM Tris, 10 mM EDTA, 100 mM NaCl, 0.1% Triton, pH 7.5) or 1 × MTSB buffer (50 mM PIPES, 5 mM MgSO_4_, and 5 mM EGTA, pH 7.2) for 5 min on ice in a vacuum and for another 25 min only on ice. After washing twice in Tris buffer or 1 × MTSB buffer, the roots were chopped in LB01 lysis buffer (15 mM Tris, 2 mM Na_2_EDTA, 0.5 mM spermine∙4HCl, 80 mM KCl, 20 mM NaCl, 15 mM β-mercaptoethanol, 0.1% (v/v) Triton X-100, pH 7.5), filtered through a 50-μm filter (CellTrics, Sysmex) and diluted 1:10, and subsequently, 100 μl of the diluted suspension was centrifuged onto microscopic slides using a Cytospin3 (Shandon, Germany) as described (Jasencakova et al. 2001). Immunostaining was performed as described by Houben et al. (2007). The following primary antibodies were used: rabbit anti-PsCENH3 (diluted 1:300), mouse anti-alpha-tubulin (clone DM 1A, Sigma, diluted 1:200), mouse anti-histone H3S10ph (Abcam, 14966, diluted 1:200), mouse anti-histone H3S28ph (Millipore, 09_797, diluted 1:200) and rabbit anti-histone H2A120ph ((Demidov et al. 2014), diluted 1:200). As secondary antibodies, a Cy3-conjugated anti-rabbit IgG (Dianova) and a FITC-conjugated anti-mouse Alexa488 antibody (Molecular Probes) were used in a 1:500 dilution each. Slides were incubated overnight at 4 °C, washed 3 times in 1 × PBS or 1 × MTSB and then the secondary antibodies were applied. Immuno-FISH was performed, according to Ishii et al. (2015).

### DNA replication analysis

Roots were treated for 2 h with 15 μM EdU (5-ethynyl-2′-deoxyuridine, baseclick GmbH), followed by water for 30 min. Preparation of slides was performed as described for immunostaining. The click reaction was performed to detect EdU according to the manual (baseclick GmbH).

### Microscopy

Images were captured using an epifluorescence microscope BX61 (Olympus) equipped with a cooled CCD camera (Orca ER, Hamamatsu). To achieve super-resolution of ~ 120 nm (with a 488-nm laser excitation), we applied spatial structured illumination microscopy (3D-SIM) using a 63x/1.40 Oil Plan-Apochromat objective of an Elyra PS.1 microscope system (Carl Zeiss GmbH) (Weisshart et al. 2016).

## Results

### *Prionium serratum* is a monocentric species

*Prionium serratum* was chosen to test whether holocentricity occurs at the base of the Cyperid clade, since this species is phylogenetically positioned at the base of a group of species recognised as holocentrics. Since the roughly 1-μm long mitotic metaphase chromosomes did not allow an unambiguous identification of a monocentromere-typical primary constriction or a holocentromere-typical parallel configuration of anaphase sister chromatids, we generated a CENH3-specific antibody suitable for immunostaining. The centromere-specific histone variant CENH3 was shown to be essential for centromere function in many species (Allshire and Karpen 2008).

First, the root transcriptome of *P. serratum* was determined, and the assembled RNAseq reads were used to identify *CENH3.* Only one *CENH3* gene was identified in the transcriptome dataset. After alignment of the corresponding amino acid sequence against CENH3s of other plant species, the evolutionary tree grouped *P. serratum* CENH3 together with other Cyperid sequences belonging to *Luzula* (Juncaceae), *Rhynchospora*, *Cyperus* and *Carex* (Cyperaceae), supporting the correct identification of the *CENH3* gene (Fig. 1).

**Fig. 2:**
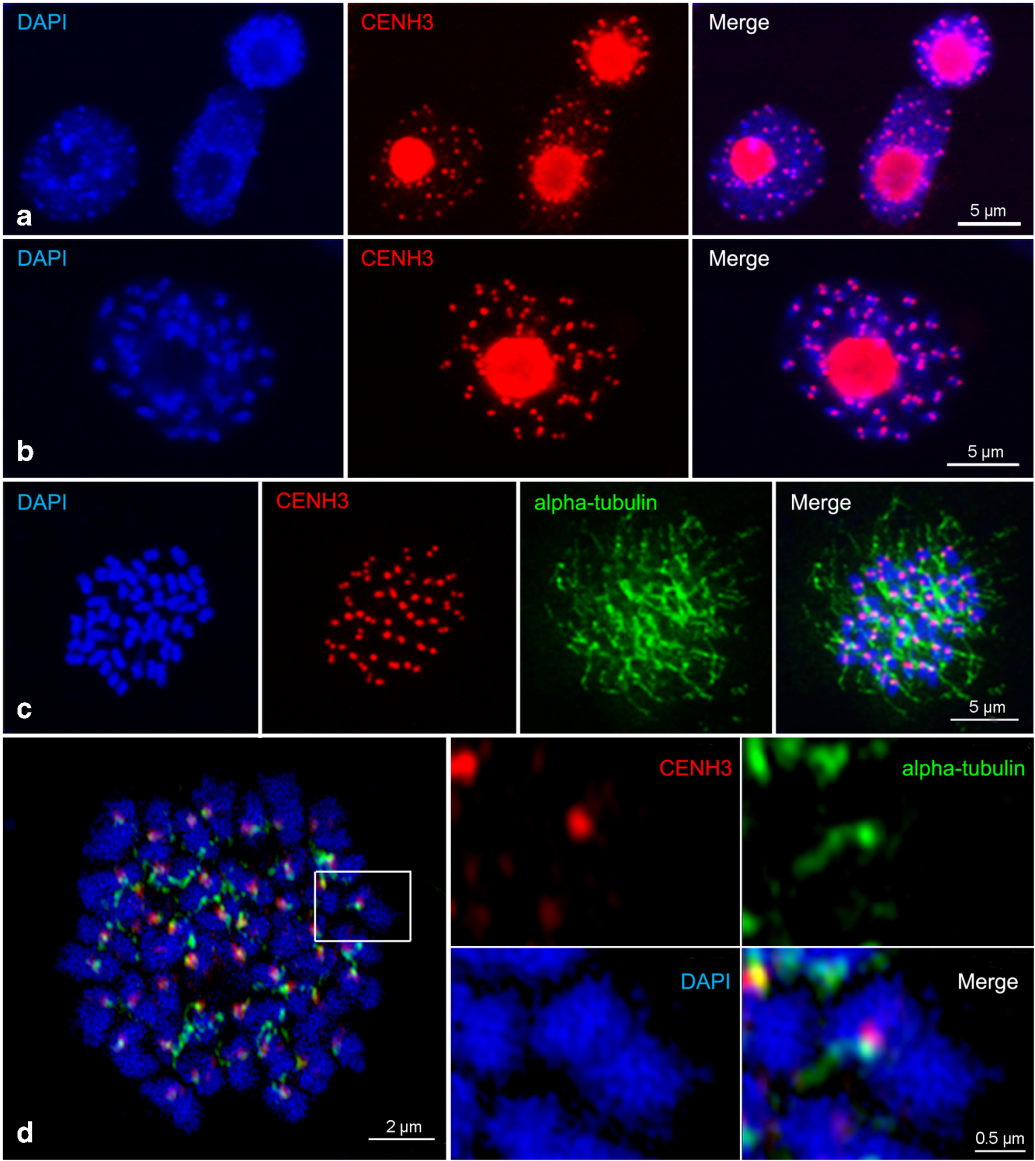
Immunodetection of centromeric protein CENH3 (red) in *P. serratum* interphase nuclei (**a**), prophase (**b**) and its interaction with alpha-tubulin (green) in metaphase chromosomes (**c**, **d**). (**d**) Image taken by spatial structured illumination microscopy (SIM), enlargement (square) shows the interaction between CENH3 and alpha-tubulin

**Fig. 3:**
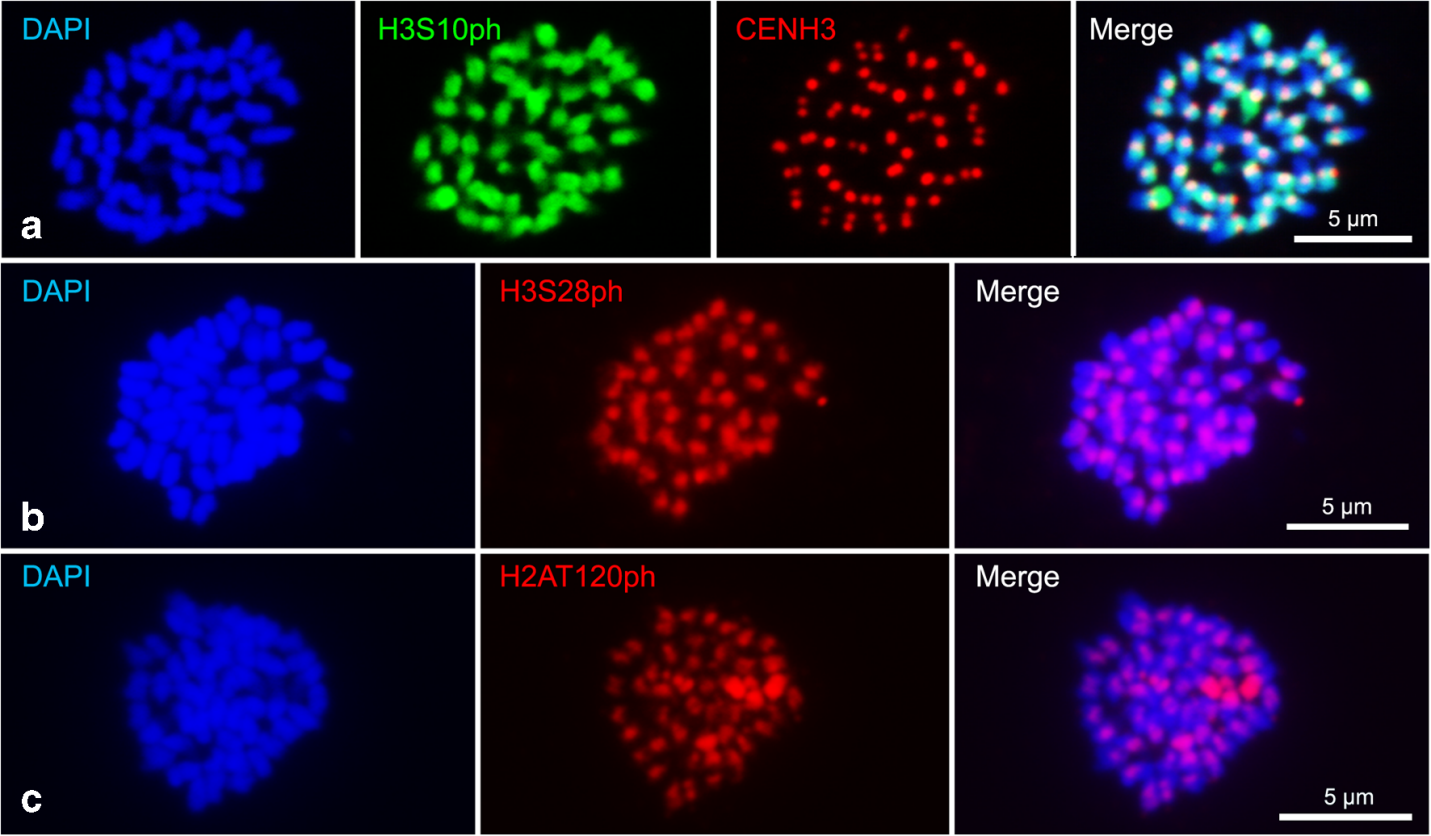
Cell cycle–dependent, pericentromere-specific histone phosphorylated modification at H3S10 (**a**), H3S28 (**b**) and H2AT120 (**c**) in metaphase chromosomes of *P. serratum*. Overlapped signals between H3S10ph (green) and CENH3 (red) are shown in (**a**)

Next, antibodies (anti-PsCENH3) designed to recognise CENH3 of *P. serratum* were generated and used for immunostaining. Typical monocentromere dot-like signals were found at interphase and at early prophase (Fig. 2a, b). Additionally, an intense labelling of the nucleoli, likely representing unspecific immunosignals, was detected. The observed interaction of CENH3 with alpha-tubulin fibres at metaphase demonstrated the centromere specificity of the CENH3 signals (Fig. 2c). The application of super-resolution microscopy confirmed the close proximity of CENH3 and tubulin signals (Fig. 2d). Besides, the cell cycle–dependent, pericentromere-specific distribution of H3S10ph, H3S28ph and H2AT120ph approved a monocentric chromosome type (Fig. 3a–c). Hence, *P. serratum* is a monocentric species based on the results obtained by the application of different (peri)centromere-specific antibodies.

### Identification of a centromere-localised repeat family *in P. serratum*

The genome size of *P. serratum* (2*n* = 46) is 335 Mbp/1C, estimated by flow cytometry. Next-generation sequence reads were generated to investigate the repetitive composition of the *P. serratum* genome based on the graph-based clustering analysis, resulting in the identification of high-copy satellite repeats and transposable elements. About 26.9% of the genome is composed of repetitive elements. The top first 329 clusters with at least 0.01% genome proportion, classified as 13 lineages of class I transposable elements (LTR retrotransposons and non-LTR LINE), six class II DNA transposons, satellite DNA (satDNA) and ribosomal DNA (rDNA) (Table 2). The LTR retrotransposons constituted ~ 9% of the genome, with the Ty1-Copia elements being more abundant than the Ty3-Gypsy elements, representing genome proportions of 5.36% and 3.63%, respectively.

**Table 2:** Repetitive families of *P. serratum*

Repeat families		Genome proportion (in %)	Total (in %)
LTR Retrotransposons			
Ty1-Copia			
	Ale	1.46	
	SIRE	1.91	
	Tork	1.68	
	Alesia	0.19	
	Ivana	0.09	
	TAR	0.02	
	Ikeros	0.01	
			5.36
Ty3-Gypsy			
	Tat	2.85	
	Chromovirus Tekay	0.42	
	Chromovirus CRM	0.20	
	Chromovirus Galadriel	0.14	
	Chromovirus Reina	0.02	
			3.63
LINE		0.28	
DNA Transposon			
	TIR	0.35	
	CACTA	0.24	
	hAT	0.35	
	MuDR Mutator	0.95	
	PIF_Harbinger	0.87	
	Helitron	0.09	
			2.85
Satellite		3.09	
rDNA			
	45S	2.68	
	5S	0.15	
Unclassified		8.89	
Total		26.93	

**Fig. 4:**
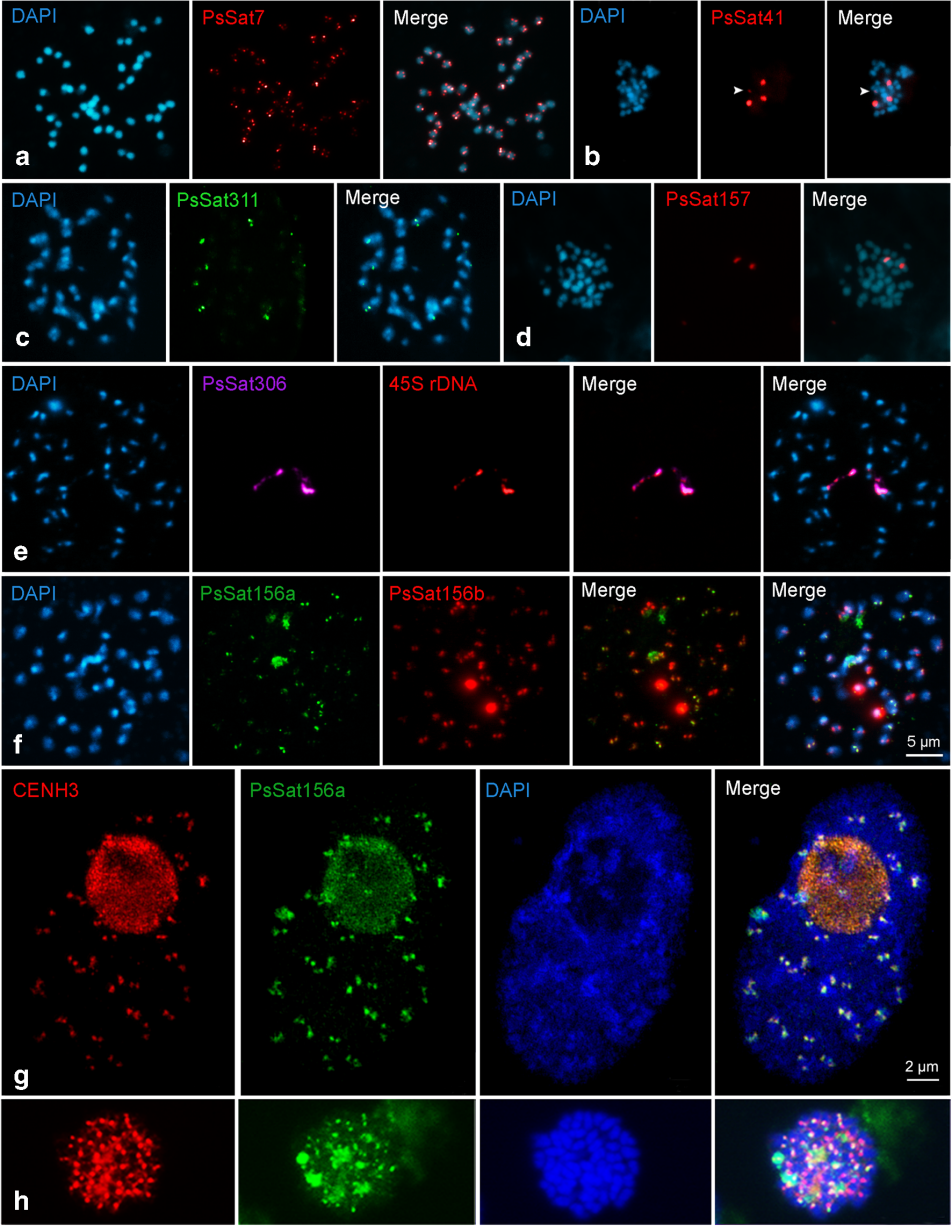
Chromosome distribution of satellite DNA families and of CENH3 in *P. serratum* (2*n* = 46). Satellite repeat PsSat7 (**a**), PsSat41 (**b**), PsSat311 (**c**), PsSat157 (**d**), PsSat306 (**e**), Ps156a and Ps156b (**f**) were mapped on metaphase chromosomes. The fourth signal of PsSat41 is indicated by arrowheads (**b**). Colocalisations between PsSat306 and 45S rDNA and between Ps156a and Ps156b are shown in (**e**) and (**f**), respectively. The centromere specificity of Ps156a was confirmed by its overlapped signals, visualised in yellow in the merge images, with CENH3 in both interphase nuclei (**g**) and metaphase chromosomes (**h**). Image (**g**) was taken by structured illumination microscopy (SIM)

The k-mer-based TAREAN analysis resulted in the identification of 19 different satDNA families. Out of these, the seven most abundant satDNAs were used for FISH to determine their chromosomal distribution. PsSat7, representing the *Arabidopsis*-type telomere sequence, hybridised to the terminal regions of all chromosomes. It is likely that the copy number of telomere repeats differs between the individual chromosome ends, as the intensity of the signals varied (Fig. 4a). PsSat41, PsSat311 and PsSat157clustered on two, four and one chromosome pairs, respectively (Fig. 4b, c, d). PsSat306 colocalised with 45S rDNA signals (Fig. 4e).

Centromere-like signals were only found after FISH with the satDNA family PsSat156 (Fig. 4f). PsSat156a and PsSat156b possess a sequence similarity of 96% but with different abundance at chromosomes. Besides dot-like signals, both probes showed enlarged hybridisation signals on one but different chromosome pairs each. To confirm the centromeric position of PsSat156a, b, immuno-FISH with the CENH3-specific antibody was performed. Colocalisation of both signals in metaphase chromosomes and interphase nuclei demonstrated the centromere specificity of the repeat family PsSat156 (Fig. 4g, Suppl. Fig. 2). No sequence similarity was found between PsSat156 and centromeric repeats of other species.

Finally, we analysed the DNA replication behaviour of *P. serratum* by 5-ethynyl-2′-deoxyuridine (EdU) incorporation, a nucleoside analogue of thymidine*.* In general, different stages of the S phase are characterized by contrasting DNA replication patterns. The early S phase was characterized by dispersed EdU signals, and clustered EdU signals were typical for the late S phase (Costas et al. 2011; Němečková et al. 2020). Whether the replication behaviour of mono- and holocentric species differs is unkown yet. However, in the holocentric species *L. elegans*, the chromosomes are less clearly compartmentalised into distinguishable early- and late-replicating chromosome regions (Heckmann et al. 2013).

**Fig. 5:**
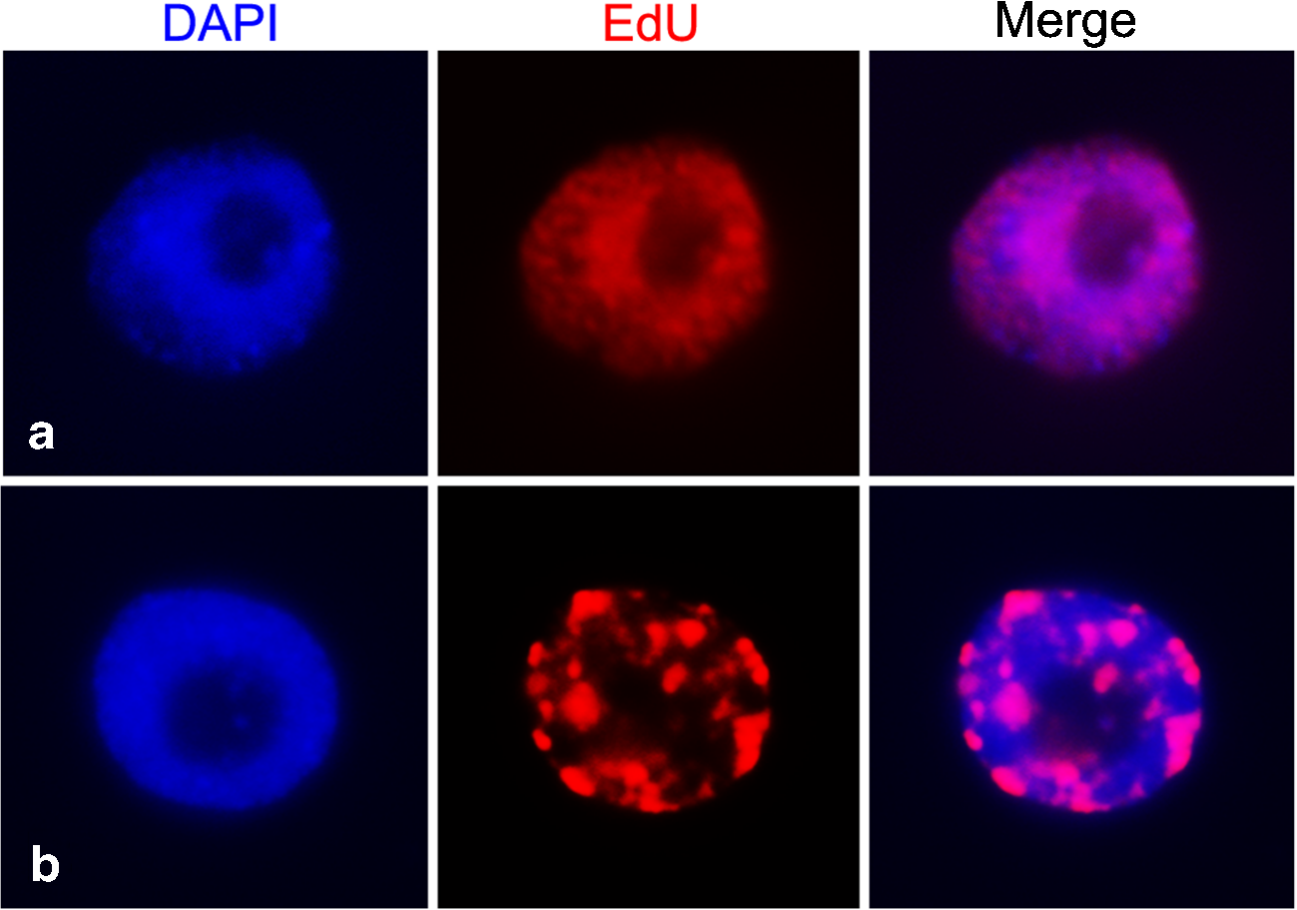
Two types of DNA replication patterns in *P. serratum* shown by EdU labelling (red) and interphase nuclei counterstained with DAPI (blue). (**a**) Mainly uniform labelling and (**b**) clustered distribution of EdU signals

The nuclei of *P. serratum* revealed two major types of labelling patterns (Fig. 5). The majority of nuclei (85% of 500 nuclei) showed an almost uniform labelling (Fig. 5a), and 15% of nuclei showed a cluster-like distribution of EdU signals (Fig. 5b). Uniformly labelled nuclei are likely at early S phase, while nuclei with clustered signals undergoing late replication. Comparable replication patterns were found in other species with monocentric chromosomes like *Arabidopsis thaliana* (Dvorackova et al. 2018) and *Zea mays* (Bass et al. 2015).

## Discussion

### Centromere evolution in Cyperids

The analysis of the centromeres by immunostaining using CENH3, alpha-tubulin, histone H3S10ph, H3S28ph and H2AT120ph antibodies demonstrated a monocentric centromere type for the phylogenetically basal *P. serratum.* Therefore, these data suggest that monocentric chromosomes may be an ancestral condition for the Juncaceae-Cyperaceae-Thurniaceae Cyperid clade. As monocentricity was also reported in species within the *Juncus* genus (Guerra et al. 2019), holocentric chromosomes in the Cyperid clade have evolved at least twice independently: once in Juncaceae and once in Cyperaceae, after the divergence of the three families. The phylogenetic close proximity of *P. serratum* CENH3 with species possessing holocentromere suggests that the sequence divergence of CENH3 does not correlate with its corresponding centromere type. A similar centromere-type independent CENH3 evolution was found for mono- and holocentric *Cuscuta* species (Oliveira et al. 2020). Hence, our data suggested that sequence modifications of CENH3 are not necessarily involved in the change of the centromere type in angiosperms.

### The abundance of repetitive DNA in *P. serratum*

The small *P. serratum* genome contains a relatively low percentage of transposable elements, ~ 9% retrotransposons and ~ 3% DNA transposons, with 13 and 6 different linages, respectively. Most plant genomes contain only a few satDNA families, mainly repeats associated with pericentromeric or subtelomeric regions (reviewed in Garrido-Ramos (2015)). Here, we identified 19 different satDNA families (~ 3% of the genome). Unlike in other small genome–sized, monocentric species, like *A. thaliana* (Maluszynska and Heslop-Harrison 1991), sugar beet (Kubis et al. 1998) and rice (Cheng et al. 2002), the centromeric satDNA is not the most abundant satDNA. PsSat306, the most abundant satDNA family, displays colocalisation with the 45S rDNA. PsSat306 likely originated from the intergenic repeat spacer region as described for other satellite repeats (reviewed in Garrido-Ramos (2015)). In addition, the 5-ethynyl-2′-deoxyuridine (EdU) detection in interphase showed comparable late replication patterns as those observed in species with monocentric chromosomes (Bass et al. 2015; Dvorackova et al. 2018).

A clustered distribution at one or only a few chromosome pairs was found for three satDNA families, similar to other satellite repeats in several species within the clade, as the holocentric *Luzula* and *Rhynchospora* genera, and outside the clade in typical monocentric species, as *Chenopodium quinoa* (Heckmann et al. 2013; Heitkam et al. 2020; Ribeiro et al. 2017). Two of these tandem repeats (PsSat156a and PsSat156b) share the same distribution at centromeric regions but with different signal intensities. Most likely, they evolved from the same ancestral centromeric repeat unit and underwent amplification or reduction at different chromosome pairs.

### How to identify holocentricity?

Results in *P. serratum* demonstrated that the characterisation of the centromere type, especially in species with small-sized chromosomes could be challenging. Which is the best method to identify holocentricity? As listed in Table 3, a range of different methods has been used to determine the centromere type in the past. However, no universal method amenable for all species exists, due to either the limitation in optical resolution, availability of specific antibodies, or required equipment.

**Table 3:** Methods to identify holocentricity

Methods	Examples	Exceptions
1. Microscopy dependent methods		
1.1 Chromosome morphology and dynamics		
1. Stable transmission of irradiation-induced chromosome fragments	• Genus *Euschistus* (Hughes-Schrader and Schrader 1961) • Genus *Chionographis* (Tanaka and Tanaka 1977)	
2. Lack of a primary constriction in mitotic metaphase chromosomes and paralleled separation of mitotic anaphase chromatids.	• *Bombyx* species (Murakami and Imai 1974) • Subgenus *Cuscuta* (Garcia 2001)	
3. In large holocentric chromosomes, sister chromatids form a distinct longitudinal centromere groove.	• Centromere groove in *Luzula elegans* (Nagaki et al. 2005; Wanner et al. 2015)	
4. Electron microscopy to determine distribution of the kinetochore plate	• *L. echinata* (Braselton 1981), *L. nivea* (Bokhari and Godward 1980) (reviewed in Mola and Papeschi, (2006), Cabral et al., (2014))	
5. Existence of inverted meiosis	• *Rhynchospora* species (Cabral et al. 2014) • *L. elegans* (Heckmann et al. 2014; Kusanagi 1962; Nordenskiold 1962)	• To deal with holocentricity during meiosis, chromosome remodelling and functional monocentricity exist in addition; e.g. temporary kinetochore activity in the end of chromosomes in kissing bug (Perez et al. 1997)
1.2 Visualise kinetochore proteins, centromere-associated histone modifications, microtubule attachment sites and rDNA loci		
1. Line-like distribution of kinetochore proteins determined by indirect immunostaining	• CENH3 signals in *L. nivea* (Nagaki et al. 2005) • CENPC signals in *R. pubera* (Marques et al. 2016)	
2. Chromosome-wide distribution of histone H3S10ph and H3S28ph and a line-like distribution of H2AT120ph, detected by indirect immunostaining	• Chromosome-wide distribution of H3S10ph and H3S28ph in *L. elegans* (Gernand et al. 2003) • Centromere-wide distribution of H2AT120ph in *L. elegans*, *L. luzuloides*, *Cyperus alternifolius* (Demidov et al. 2014) and *R. pubera* (Cabral et al. 2014)	
3. Attachment of alpha-tubulin fibres along the entire length of chromosomes by indirect immunostaining	• *L .elegans* (Heckmann et al. 2014; Heckmann et al. 2011; Nagaki et al. 2005) • *C. europea* (Oliveira et al. 2020)	
4. Terminal position of 45S rDNA loci determined by FISH	• Terminal position of 45S rDNA in 42 holocentric plant species (reviewed in Roa and Guerra (2012))	• Interstitial 45S rDNA in holocentric Lepidoptera (Nguyen et al. 2010)
2. Microscopy-independent methods: flow cytometry and assessment of genomic content		
1. The proportion of G2 nuclei determined by flow cytometry after radiation-induced fragmentation	• A strongly elevated proportion of G2 nuclei in monocentric species (Zedek et al. 2016)	• *Prionium serratum* (current study)
2. Genomic GC content	• Dramatic decreases in GC content in holocentric species (Smarda et al. 2014)	• *P. serratum* (current study)

Cytological methods, by observing the absence of a primary constriction in mitotic chromosomes, paralleled segregation of anaphase sister chromatids and the faithful transmission of induced chromosomal fragments, are the prime methods of choice to identify holocentrics. In large chromosome species like *L. elegans* and *R. pubera*, holocentromeres form at somatic pro- and metaphase a distinct longitudinal groove along each sister chromatid which is visible by standard (Heckmann et al. 2011; Nagaki et al. 2005), structured illumination and scanning electron microscopy (Marques et al. 2015; Wanner et al. 2015).

However, the first two methods are not applicable for small chromosome species. The analysis of irradiation-induced chromosome fragments is still one of the best methods to verify holocentricity (Hughes-Schrader and Ris 1941; reviewed in Mola and Papeschi (2006)). While acentric fragments of monocentric chromosomes form micronuclei, induced holocentric fragments are stably transmitted into the next cell generation and do not form micronuclei. But the application of this method requires specialised equipment for the generation of ionising radiation.

The analysis of meiotic chromosome dynamics has been used to determine holocentricity in species with moderate to large chromosomes (reviewed in Cuacos et al. 2015; Marques and Pedrosa-Harand 2016). Three principle options exist to deal with holocentricity during meiosis: (i) ‘chromosome remodelling’, (ii) ‘functional monocentricity’ and (iii) ‘inverted meiosis’. In the case of inverted meiosis, in contrast to monopolar sister centromere orientation, the unfused holokinetic sister centromeres behave as two distinct functional units during meiosis I, resulting in sister chromatid separation. Homologous non-sister chromatids remain terminally linked by a hardly visible chromatin fibre. Then, they separate at anaphase II. Thus, an inverted sequence of meiotic sister chromatid segregation occurs.

An almost terminal position of 45S rDNA, adjacent to telomeres, has been linked to holocentricity. This observation was made in 42 species of seven genera with holokinetic chromosomes (Roa and Guerra 2012). A possible explanation is that a secondary constriction in the interstitial region would interrupt the kinetochore plate along the holokinetic chromosome establishing a condition similar to dicentric chromosomes, leading to errors in chromosome segregation (Heckmann et al. 2011). But in holocentric *Lepidoptera* species also interstitial 45S rDNA sites were detected (Nguyen et al. 2010). Thus, since the terminal 45S rDNA location is not universal in holocentrics, it is not a universal evidence for holocentricity. Also, a terminal position of 45S rDNA was found in monocentric species (Schubert and Wobus 1985).

Visualisation of kinetochore proteins, such as CENH3 or CENPC, by immunodetection shows the centromere type directly (Marques et al. 2016; Nagaki et al. 2005). This strategy is less restricted by chromosome size. However, it is often limited by the availability of species-specific kinetochore antibodies, which are both time- and cost-consuming in production. However, the absence of CENH3 in some species (Drinnenberg et al. 2014) and the microtubule attachment at CENH3-free chromosome regions in some species (Oliveira et al. 2020) make the application of anti-CENH3 as a universal marker for centromeres questionable. Nevertheless, the analysis of kinetochore proteins could be complemented by combining the investigation of the spindle fibre attachment using alpha-tubulin-specific antibodies if the size of chromosomes allows the identification of the spindle fibre attachment site. In addition, the application of antibodies specific for the cell cycle–dependent pericentromeric phosphorylation of histone H3 (H3S10ph, H3S28ph) and H2A (H2AT120ph) resulted in the identification of holocentromere-specific immunostaining patterns (Demidov et al. 2014; Gernand et al. 2003). In monocentric plants, immunostaining with antibodies against H3S10ph and H3S28ph results in a specific labelling of the pericentromere in mitotic chromosomes. In contrast, in holocentric plants, immunolabelling with the same antibodies results in uniform staining of condensed chromosomes, due to the chromosome-wide distribution of the pericentromere (Gernand et al. 2003). The application of these antibodies in a wide range of species is possible due to the evolutionarily conserved amino acid sequence of histone H3. However, in some monocentric species, the application of anti-H2AT120ph resulted in additional non-pericentromeric signals (Baez et al. 2019; Sousa et al. 2016).

Transmission electron microscopy studies also showed differences between holo- and monocentric chromosomes in relation to the size and distribution of the kinetochore plate (reviewed in Mola and Papeschi (2006)). However, the preparation of specimens for electron microscopy is somewhat laborious, and therefore, it is less suitable for routine work.

Microscopy-independent flow cytometry and sequence-based approaches, by analysing irradiation-induced G2 nuclei accumulation and GC content, respectively, were developed for identifying the centromere type (Smarda et al. 2014; Zedek et al. 2016). But, as our analysis of *P. serratum* showed, indirect methods should be taken with care. Hence, as no universal and straightforward method exists, if possible, different techniques should be combined to determine holocentricity, depending on the characteristics for each particular species.

## Supplementary Information

ESM 1 (DOCX 248 kb)

## References

[CR1] Aliyeva-Schnorr L, Beier S, Karafiatova M, Schmutzer T, Scholz U, Dolezel J, Stein N, Houben A (2015). Cytogenetic mapping with centromeric bacterial artificial chromosomes contigs shows that this recombination-poor region comprises more than half of barley chromosome 3H. Plant J.

[CR2] Allshire RC, Karpen GH (2008). Epigenetic regulation of centromeric chromatin: old dogs, new tricks?. Nat Rev Genet.

[CR3] Andrews S (2010) FastQC: A quality control tool for high throughput sequence data [Online]. Available online at: http://www.bioinformatics.babraham.ac.uk/projects/fastqc/

[CR4] Baez M, Vaio M, Dreissig S, Schubert V, Houben A, Pedrosa-Harand A (2019). Together but different: the subgenomes of the bimodal *Eleutherine* karyotypes are differentially organized. Front Plant Sci.

[CR5] Bass HW, Hoffman GG, Lee TJ, Wear EE, Joseph SR, Allen GC, Hanley-Bowdoin L, Thompson WF (2015). Defining multiple, distinct, and shared spatiotemporal patterns of DNA replication and endoreduplication from 3D image analysis of developing maize (*Zea mays* L.) root tip nuclei. Plant Mol Biol.

[CR6] Bokhari FS, Godward MBS (1980). The ultrastructure of the diffuse kinetochore in *Luzula nivea*. Chromosoma.

[CR7] Bolger AM, Lohse M, Usadel B (2014). Trimmomatic: a flexible trimmer for Illumina sequence data. Bioinformatics.

[CR8] Braselton JP (1981). The ultrastructure of meiotic kinetochores of *Luzula*. Chromosoma.

[CR9] Bures P, Zedek F, Markova M (2012) Holocentric chromosomes. book chapter: J- Greilhuber et al. (eds) Plant genome diversity, Volume 1, Springer Verlag Wien

[CR10] Cabral G, Marques A, Schubert V, Pedrosa-Harand A, Schlogelhofer P (2014). Chiasmatic and achiasmatic inverted meiosis of plants with holocentric chromosomes. Nat Commun.

[CR11] Cheng Z, Dong F, Langdon T, Ouyang S, Buell CR, Gu M, Blattner FR, Jiang J (2002). Functional rice centromeres are marked by a satellite repeat and a centromere-specific retrotransposon. Plant Cell.

[CR12] Cock PJA, Chilton JM, Grüning B, Johnson JE, Soranzo N (2015). NCBI BLAST+ integrated into Galaxy. GigaScience.

[CR13] Costas C, Sanchez Mde L, Sequeira-Mendes J, Gutierrez C (2011). Progress in understanding DNA replication control. Plant Sci.

[CR14] Cuacos M, Franklin FC, Heckmann S (2015). Atypical centromeres in plants - what they can tell us. Front Plant Sci.

[CR15] Demidov D, Schubert V, Kumke K, Weiss O, Karimi-Ashtiyani R, Buttlar J, Heckmann S, Wanner G, Dong Q, Han F, Houben A (2014). Anti-phosphorylated histone H2AThr120: a universal microscopic marker for centromeric chromatin of mono- and holocentric plant species. Cytogenet Genome Res.

[CR16] Dernburg AF (2001). Here, there, and everywhere: kinetochore function on holocentric chromosomes. J Cell Biol.

[CR17] Dolêzel J, Bartos J, Voglmayr H, Greilhuber J (2003). Nuclear DNA content and genome size of trout and human. Cytometry A.

[CR18] Dong Q, Han F (2012). Phosphorylation of histone H2A is associated with centromere function and maintenance in meiosis. Plant J.

[CR19] Drinnenberg IA, deYoung D, Henikoff S, Malik HS (2014). Recurrent loss of CenH3 is associated with independent transitions to holocentricity in insects. Elife.

[CR20] Dvorackova M, Raposo B, Matula P, Fuchs J, Schubert V, Peska V, Desvoyes B, Gutierrez C, Fajkus J (2018). Replication of ribosomal DNA in *Arabidopsis* occurs both inside and outside the nucleolus during S phase progression. J Cell Sci.

[CR21] Escudero M, Marquez-Corro JI, Hipp AL (2016). The phylogenetic origins and evolutionary history of holocentric chromosomes. Syst Bot.

[CR22] Fu L, Niu B, Zhu Z, Wu S, Li W (2012). CD-HIT: accelerated for clustering the next-generation sequencing data. Bioinformatics.

[CR23] Garcia MA (2001). A new western Mediterranean species of *Cuscuta* (Convolvulaceae) confirms the presence of holocentric chromosomes in subgenus *Cuscuta*. Bot J Linn Soc.

[CR24] Garrido-Ramos MA (2015). Satellite DNA in plants: more than just rubbish. Cytogenet Genome Res.

[CR25] Gerlach WL, Bedbrook JR (1979). Cloning and characterization of ribosomal RNA genes from wheat and barley. Nucleic Acids Res.

[CR26] Gernand D, Demidov D, Houben A (2003). The temporal and spatial pattern of histone H3 phosphorylation at serine 28 and serine 10 is similar in plants but differs between mono- and polycentric chromosomes. Cytogenet Genome Res.

[CR27] Greilhuber J (1995) Chromosomes of the moncotyledons (General aspects). In P.J. Rudall, P.J. Cribb. D.f. Cutler & C.J. Humphries (Eds.) Moncotyledons: systematcs and evolution, pp. 370-414. Royal Botanic Gardens, Kew, 379-414

[CR28] Guerra M, Ribeiro T, Felix LP (2019). Monocentric chromosomes in *Juncus* (Juncaceae) and implications for the chromosome evolution of the family. Bot J Linn Soc.

[CR29] Heckmann S, Schroeder-Reiter E, Kumke K, Ma L, Nagaki K, Murata M, Wanner G, Houben A (2011). Holocentric chromosomes of *Luzula elegans* are characterized by a longitudinal centromere groove, chromosome bending, and a terminal nucleolus organizer region. Cytogenet Genome Res.

[CR30] Heckmann S, Macas J, Kumke K, Fuchs J, Schubert V, Ma L, Novak P, Neumann P, Taudien S, Platzer M, Houben A (2013). The holocentric species *Luzula elegans* shows interplay between centromere and large-scale genome organization. Plant J.

[CR31] Heckmann S, Jankowska M, Schubert V, Kumke K, Ma W, Houben A (2014). Alternative meiotic chromatid segregation in the holocentric plant *Luzula elegans*. Nature Commu.

[CR32] Heitkam T, Weber B, Walter I, Liedtke S, Ost C, Schmidt T (2020). Satellite DNA landscapes after allotetraploidization of quinoa (*Chenopodium quinoa*) reveal unique A and B subgenomes. Plant J.

[CR33] Hochbach A, Linder HP, Roser M (2018). Nuclear genes, matK and the phylogeny of the Poales. Taxon.

[CR34] Houben A, Schroeder-Reiter E, Nagaki K, Nasuda S, Wanner G, Murata M, Endo TR (2007). CENH3 interacts with the centromeric retrotransposon cereba and GC-rich satellites and locates to centromeric substructures in barley. Chromosoma.

[CR35] Huang YC, Lee CC, Kao CY, Chang NC, Lin CC, Shoemaker D, Wang J (2016). Evolution of long centromeres in fire ants. BMC Evol Biol.

[CR36] Hughes-Schrader S, Ris H (1941). The diffuse spindle attachment of coccids, verified by the mitotic behavior of induced chromosome fragments. J Exp Zool.

[CR37] Hughes-Schrader S, Schrader F (1961). The kinetochore of the Hemiptera. Chromosoma.

[CR38] Ishii T, Karimi-Ashtiyani R, Banaei-Moghaddam AM, Schubert V, Fuchs J, Houben A (2015). The differential loading of two barley CENH3 variants into distinct centromeric substructures is cell type- and development-specific. Chromosom Res.

[CR39] Jankowska M, Fuchs J, Klocke E, Fojtova M, Polanska P, Fajkus J, Schubert V, Houben A (2015). Holokinetic centromeres and efficient telomere healing enable rapid karyotype evolution. Chromosoma.

[CR40] Jasencakova Z, Meister A, Schubert I (2001). Chromatin organization and its relation to replication and histone acetylation during the cell cycle in barley. Chromosoma.

[CR41] Judd WS, Campbell CS, Kellogg EA, Stevens PF, Donoghue MJ (2016) Plant systematics: a phylogenetic approach. 4th. MA Sunderland: Sinauer Associates

[CR42] Kubis S, Schmidt T, Heslop-Harrison JS (1998). Repetitive DNA elements as a major component of plant genomes. Ann Bot.

[CR43] Kumar S, Stecher G, Suleski M, Hedges SB (2017). TimeTree: a resource for timelines, timetrees, and divergence times. Mol Biol Evol.

[CR44] Kumar S, Stecher G, Li M, Knyaz C, Tamura K (2018). MEGA X: Molecular Evolutionary Genetics Analysis across computing platforms. Mol Biol Evol.

[CR45] Kusanagi A (1962). Mechanism of post-reductional meiosis in *Luzula*. Jpn J Genet.

[CR46] Letunic I, Bork P (2007). Interactive Tree Of Life (iTOL): an online tool for phylogenetic tree display and annotation. Bioinformatics.

[CR47] Letunic I, Bork P (2019). Interactive Tree Of Life (iTOL) v4: recent updates and new developments. Nucleic Acids Res.

[CR48] Li W, Godzik A (2006). Cd-hit: a fast program for clustering and comparing large sets of protein or nucleotide sequences. Bioinformatics.

[CR49] Maluszynska J, Heslop-Harrison JS (1991). Localization of tandemly repeated DNA sequences in *Arabidopsis thaliana*. Plant J.

[CR50] Marques A, Pedrosa-Harand A (2016). Holocentromere identity: from the typical mitotic linear structure to the great plasticity of meiotic holocentromeres. Chromosoma.

[CR51] Marques A, Ribeiro T, Neumann P, Macas J, Novak P, Schubert V, Pellino M, Fuchs J, Ma W, Kuhlmann M, Brandt R, Vanzela AL, Beseda T, Simkova H, Pedrosa-Harand A, Houben A (2015). Holocentromeres in *Rhynchospora* are associated with genome-wide centromere-specific repeat arrays interspersed among euchromatin. Proc Natl Acad Sci U S A.

[CR52] Marques A, Schubert V, Houben A, Pedrosa-Harand A (2016). Restructuring of holocentric centromeres during meiosis in the plant *Rhynchospora pubera*. Genetics.

[CR53] Marquez-Corro JI, Escudero M, Luceno M (2018). Do holocentric chromosomes represent an evolutionary advantage? A study of paired analyses of diversification rates of lineages with holocentric chromosomes and their monocentric closest relatives. Chromosom Res.

[CR54] Melters DP, Paliulis LV, Korf IF, Chan SW (2012). Holocentric chromosomes: convergent evolution, meiotic adaptations, and genomic analysis. Chromosom Res.

[CR55] Mola LM, Papeschi AG (2006). Holokinetic chromsomes at a glance. J Basic Appl Genet.

[CR56] Murakami A, Imai HT (1974). Cytological evidence for holocentric chromosomes of silkworms, *Bombyx mori* and *B. mandarina*, (*Bombycidae*, *Lepidoptera*). Chromosoma.

[CR57] Nagaki K, Kashihara K, Murata M (2005). Visualization of diffuse centromeres with centromere-specific histone H3 in the holocentric plant Luzula nivea. Plant Cell.

[CR58] Němečková A, Koláčková V, Vrána J, Doležel D, Hřibová E (2020) DNA replication and chromosome positioning throughout the interphase in three-dimensional space of plant nuclei. J Exp Bot (in press):eraa370. 10.1093/jxb/eraa370 10.1093/jxb/eraa370 32805034

[CR59] Neumann P, Navratilova A, Schroeder-Reiter E, Koblizkova A, Steinbauerova V, Chocholova E, Novak P, Wanner G, Macas J (2012). Stretching the rules: monocentric chromosomes with multiple centromere domains. PLoS Genet.

[CR60] Neumann P, Schubert V, Fukova I, Manning JE, Houben A, Macas J (2016). Epigenetic histone marks of extended meta-polycentric centromeres of Lathyrus and Pisum chromosomes. Front Plant Sci.

[CR61] Nguyen P, Sahara K, Yoshido A, Nguyen P, Sahara K, Yoshido A, Marec F (2010). Evolutionary dynamics of rDNA clusters on chromosomes of moths and butterflies (Lepidoptera). Genetica.

[CR62] Nordenskiold H (1962). Studies of meiosis in *Luzula purpurea*. Hereditas.

[CR63] Novak P, Neumann P, Pech J, Steinhaisl J, Macas J (2013). RepeatExplorer: a Galaxy-based web server for genome-wide characterization of eukaryotic repetitive elements from next-generation sequence reads. Bioinformatics.

[CR64] Novak P, Avila Robledillo L, Koblizkova A, Vrbova I, Neumann P, Macas J (2017). TAREAN: a computational tool for identification and characterization of satellite DNA from unassembled short reads. Nucleic Acids Res.

[CR65] Oliveira L, Neumann P, Jang T-S, Klemme S, Schubert V, Koblížková A, Houben A, Macas J (2020). Mitotic spindle attachment to the holocentric chromosomes of *Cuscuta europaea* does not correlate with the distribution of CENH3 chromatin. Front Plant Sci.

[CR66] Perez R, Panzera F, Page J, Suja JA, Rufas JS (1997). Meiotic behaviour of holocentric chromosomes: orientation and segregation of autosomes in *Triatoma infestans* (Heteroptera). Chromosom Res.

[CR67] Ribeiro T, Marques A, Novak P, Schubert V, Vanzela AL, Macas J, Houben A, Pedrosa-Harand A (2017). Centromeric and non-centromeric satellite DNA organisation differs in holocentric *Rhynchospora* species. Chromosoma.

[CR68] Roa F, Guerra M (2012). Distribution of 45S rDNA sites in chromosomes of plants: structural and evolutionary implications. BMC Evol Biol.

[CR69] Schubert I, Wobus U (1985). *In situ* hybridisation confirms jumping nucleolus organizing regions in *Allium*. Chromosoma.

[CR70] Schubert V, Neumann P, Marques A, Heckmann S, Macas J, Pedrosa-Harand A, Schubert I, Jang TS, Houben A (2020). Super-resolution microscopy reveals diversity of plant centromere architecture. Int J Mol Sci.

[CR71] Semmouri I, Bauters K, Leveille-Bourret E, Starr JR, Goetghebeur P, Larridon I (2019). Phylogeny and systematics of Cyperaceae, the evolution and importance of embryo morphology. Bot Rev.

[CR72] Sheikh SA, Kondo K, Hoshi Y (1995). Study on diffused centromeric nature of *Drosera* chromosomes. Cytologia.

[CR73] Silva AD, Alves MVS, Coan AI (2020). Comparative floral morphology and anatomy of Thurniaceae, an early-diverging family in the cyperids (Poales, Monocotyledons). Plant Syst Evol.

[CR74] Smarda P, Bures P, Horova L, Leitch IJ, Mucina L, Pacini E, Tichy L, Grulich V, Rotreklova O (2014). Ecological and evolutionary significance of genomic GC content diversity in monocots. Proc Natl Acad Sci U S A.

[CR75] Smith-Unna R, Boursnell C, Patro R, Hibberd JM, Kelly S (2016). TransRate: reference-free quality assessment of *de novo* transcriptome assemblies. Genome Res.

[CR76] Sousa A, Bellot S, Fuchs J, Houben A, Renner SS (2016). Analysis of transposable elements and organellar DNA in male and female genomes of a species with a huge Y chromosome reveals distinct Y centromeres. Plant J.

[CR77] Tanaka N, Tanaka N (1977). Chromosome studies in Chionographis (Liliaceae) I. Holokinetic nature of chromosomes in *Chionographis japonica* maxim. Cytologia.

[CR78] Thompson JD, Higgins DG, Gibson TJ (1994). Clustal W: improving the sensitivity of progressive multiple sequence alignment through sequence weighting, position-specific gap penalties and weight matrix choice. Nucleic Acids Res.

[CR79] Trifinopoulos J, Nguyen LT, von Haeseler A, Minh BQ (2016). W-IQ-TREE: a fast online phylogenetic tool for maximum likelihood analysis. Nucleic Acids Res.

[CR80] Wanner G, Schroeder-Reiter E, Ma W, Houben A, Schubert V (2015). The ultrastructure of mono- and holocentric plant centromeres: an immunological investigation by structured illumination microscopy and scanning electron microscopy. Chromosoma.

[CR81] Weisshart K, Fuchs J, Schubert V (2016). Structured illumination microscopy (SIM) and photoactivated localization microscopy (PALM) to analyze the abundance and distribution of RNA polymerase II molecules on flow-sorted Arabidopsis nuclei. Bio-protocol.

[CR82] Zedek F, Bures P (2018). Holocentric chromosomes: from tolerance to fragmentation to colonization of the land. Ann Bot.

[CR83] Zedek F, Vesely P, Bures P (2016). Flow cytometry may allow microscope-independent detection of holocentric chromosomes in plants. Sci Rep.

