# Supplementary material for: Analysis of the small chromosomal Prionium serratum (Cyperid) demonstrates the importance of reliable methods to differentiate between mono- and holocentricity
Source: Chromosoma. 2020 Nov 9;129(3):285–97. doi: 10.1007/s00412-020-00745-6 (PMC7665975; doi:10.1007/s00412-020-00745-6)
Supplement: Supplementary file 1 — (DOCX 248 kb) [file 412_2020_745_MOESM1_ESM.docx]

Supplementary Material


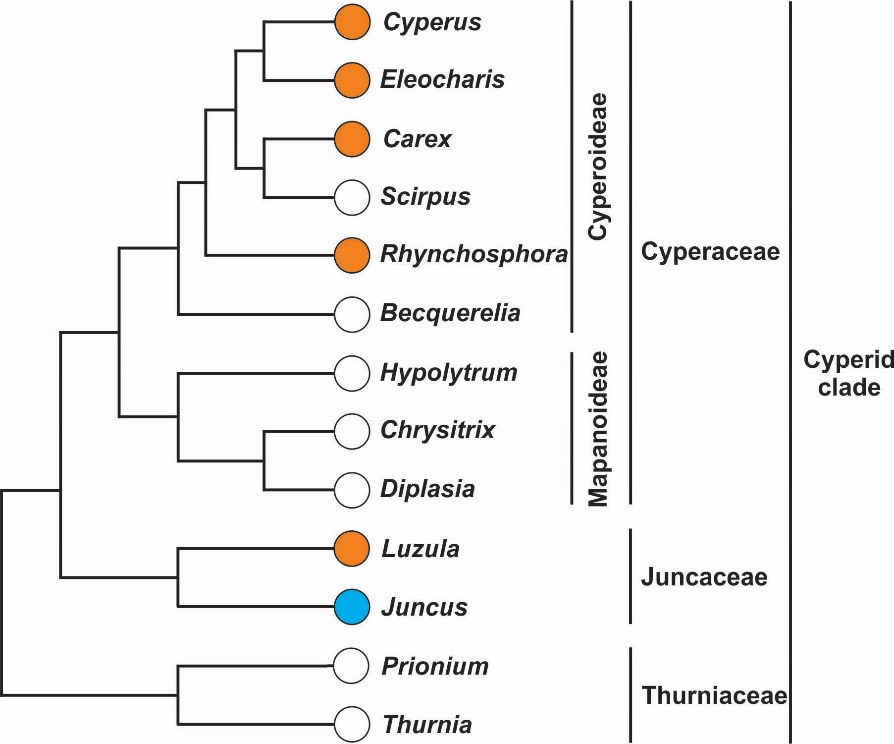


Suppl. Figure 1

Phylogenetic relationship of the genera within the Cyperid clade. Confirmed holocentric genera are labelled by an orange circle; monocentric ones with a blue circle; genera with no centromere information with an empty circle. Phylogeny simplified from Hochbach *et al.* (2018), Semmouri *et al.* (2019), Silva *et al.*, (2020).


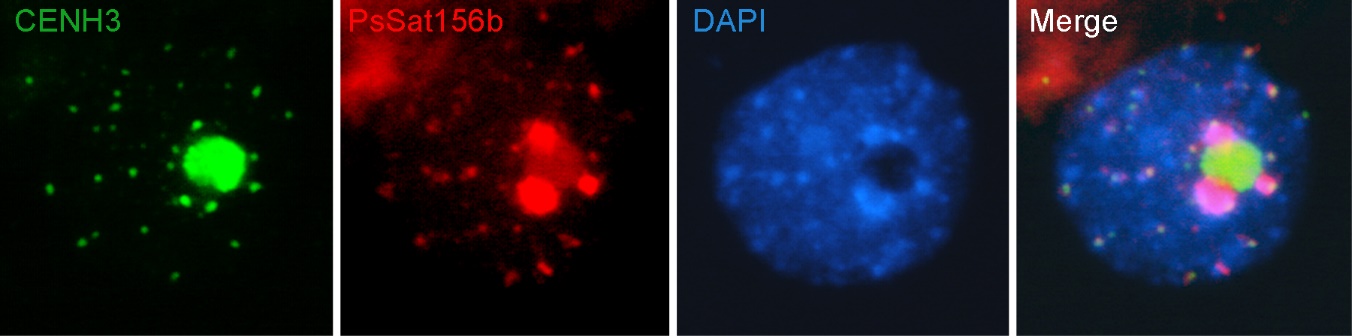


Suppl. Figure 2

Localisation of CENH3 and PsSat156b satellite repeat signals in *P. serratum* interphase nucleus. The centromere positioning of PsSat156b is confirmed by its colocalisation with CENH3. Additional strong PsSat156b signals, also shown on metaphase chromosomes (Figure 4F), locate at the peripheral of the nucleolus.

Suppl. Table 1 NCBI accession numbers of plant CENH3 sequences used in the phylogenetic analysis

| Accession number | Plant histone CENH3 or H3 |
| --- | --- |
| QGY64356 | *Cuscuta europaea 1A* |
| QGY64360 | *Cuscuta europaea 2* |
| QGY64362 | *Cuscuta campestris A* |
| QGY64361 | *Cuscuta campestris B* |
| QGW49108 | *Cyperus fuscus* |
| QGW49107 | *Cyperus textilis* |
| QGW49119 | *Carex humilis* |
| QGW49120 | *Carex caryophyllea* |
| XP 010931498 | *Elaeis guineensis X2* |
| XP 019708718 | *Elaeis guineensis X1* |
| XP 008792454 | *Phoenix dactylifera* |
| AMH40810 | *Musa balbisiana* |
| AKI32604 | *Musa acuminata* |
| XP 020113817 | *Ananas comosus* |
| BAL45431 | *Allium tuberosum* |
| BAL45432 | *Allium cepa* |
| BAL45430 | *Allium sativum* |
| ACX30889 | *Oryza alta* |
| ACX30893 | *Oryza australiensis* |
| AKM28569 | *Aegilops tauschii* |
| AEH95350 | *Triticum aestivum* |
| ALF04640 | *Rhynchospora pubera 2* |
| ALF04639 | *Rhynchospora pubera 1* |
| NP 001105520 | *Zea mays* |
| AOR06534 | *Luzula elegans 1* |
| AOR06535 | *Luzula elegans 2* |
| ADM18965 | *Luzula nivea* |
| XP 020572267 | *Phalaenopsis equestris X1* |
| XP 020572268 | *Phalaenopsis equestris X2* |
| ACZ04978 | *Brassica nigra* |
| NP 001288957 | *Brassica rapa* |
| BAF49733 | *Raphanus sativus* |
| AAL86775 | *Arabidopsis thaliana* |
| AAT96392 | *Arabidopsis lyrata* |
| NP 001289450 | *Nicotiana tomentosiformis* |
| NP 001289496 | *Nicotiana sylvestris* |
| AID21730 | *Daucus pusillus* |
| AID21731 | *Daucus glochidiatus* |
| XP 007219168 | *Prunus persica* |
| XP 021815253 | *Prunus avium* |
| XP 023754490 | *Lactuca sativa* |
| XP 006339687 | *Solanum tuberosum* |
| XP 010326926 | *Solanum lycopersicum* |
| XP 010661899 | *Vitis vinifera 1* |
| XP 002281073 | *Vitis vinifera 2* |
| XP 011659153 | *Cucumis sativus* |
| XP 022156549 | *Momordica charantia* |
| XP 022959605 | *Cucurbita moschata* |
| XP 022012308 | *Helianthus annuus* |
| BAF49727 | *Crucihimalaya wallichii* |
| AAT96391 | *Crucihimalaya himalaica* |
| AUN88469 | *Secale sylvestre beta* |
| AUN88454 | *Secale sylvestre alpha* |
| PKA51165 | *Apostasia shenzhenica* |
| AAA32809 | *Arabidopsis thaliana H3** |
| ADI87407 | *Oryza sativa H3** |

*The canonical histone H3 sequences were used as outgroup
